# Self-reported health-related quality of life (HRQoL) and factors affecting HRQoL among individuals with health insurance in Iran

**DOI:** 10.4178/epih.e2016046

**Published:** 2016-10-26

**Authors:** Ali Kazemi Karyani, Arash Rashidian, Sarar Emamgholipour Sefiddashti, Ali Akbari Sari

**Affiliations:** Department of Health Management and Economics, School of Public Health, Tehran University of Medical Sciences, Tehran, Iran

**Keywords:** Health related quality of life, Quality of life, Health, Insurance, Iran

## Abstract

**OBJECTIVES:**

The aim of this study was to measure the health-related quality of life (HRQoL) and to evaluate the factors affecting HRQoL in individuals with health insurance in Tehran, Iran.

**METHODS:**

A cross-sectional analytical study was conducted using the 3-level EuroQol 5-dimensions (EQ-5D) questionnaire. In order to estimate the determinants of HRQoL, information about participants’ demographic, socioeconomic, and health status was gathered. The cluster sampling technique was used to collect data from May to June, 2016. The chi-square test and weighted least squares method were employed for data analysis. Data were analyzed using Stata version 11.0.

**RESULTS:**

A total of 600 Iranians with insurance completed the study, of whom 327 (54.5%) were male and 273 (45.5%) were female. The mean age of the participants was 41.48 years (standard deviation [SD], 14.60 years). Meanwhile, the mean duration of education was 12.36 years (SD, 4.68 years). The mean EQ-5D score was 0.74 (SD, 0.16). The most common health problems in the participants were anxiety/depression (42.3%), followed by pain/discomfort (39.2%). Sex, age, years of schooling, income, chronic disease, and body mass index had a significant effect on HRQoL (p<0.05). Healthy insured individuals, on average, had a HRQoL score 0.119 higher than that of people with a chronic disease, all else being equal (p<0.001).

**CONCLUSIONS:**

Among all determinants of HRQoL, chronic disease was found to be the highest priority for interventions to improve the health status of Iranians with insurance. This finding can help policymakers and health insurance organizations improve their planning to promote the HRQoL of individuals with insurance and society as a whole in Iran.

## INTRODUCTION

Health-related quality of life (HRQoL) is a measure that reflects individuals’ subjective experiences of their health status. HRQoL systematically focuses on measuring the relationship between health and health status with quality of life (QoL) [1,2]. It is a dynamic multidimensional model and it consists of three main dimensions: the physical, social, and mental dimensions of health [3].

In order to improve the QoL in various populations and to reduce health inequalities, it is important to note that certain domains and the factors related to those domains might be especially influential for subjects’ HRQoL [3,4]. Therefore, many researchers have examined HRQoL and the factors affecting population health and health-related conditions, based on the hypothesis that this is an optimal way to characterize the severity of these problems [5-7]. However, HRQoL has not been thoroughly characterized among those with health insurance. Health insurance coverage has a tremendous impact on access to healthcare services. Moreover, people with health insurance have better physical and mental health than those who are not insured [8-10].

In Iran, the health system is insurance-based, which has had an important effect on the healthcare system in the country [11]. The Social Security Organization, which was renamed the Social Security Insurance Organization (SSIO) in 1980, has provided health insurance coverage for almost all Iranian employees since 1975. Subsequently, the Medical Services Insurance Organization, which is currently known as the Iran Health Insurance Organization (IHIO), was established in 1994 in order to provide health insurance coverage for both governmental employees and those who had not yet been insured [11]. More than 1,670,000 people have IHIO insurance in Tehran city. Meanwhile, approximately six million people have SSIO insurance in Tehran city [12,13]. Evidence has been published regarding the HRQoL of patients with many diseases and chronic conditions in Iran [14,15]. Nonetheless, insufficient evidence-based information regarding HRQoL and its determinants within the essential health and health-related insurance system in Tehran and throughout Iran has been published. Given the lack of key evidence for policymakers in the country, we were selected all individuals with insurance as the study population. Most Iranians have health insurance, and we chose to focus on the insured people because they comprise a representative subset of the population in Tehran. Therefore, the aim of this study was to measure HRQoL and determine factors related to HRQoL in individuals with basic health insurance in Tehran, Iran. We additionally examined the dimensions of health according to age.

## MATERIALS AND METHODS

This study was conducted in Tehran, the capital city of Iran. A cross-sectional analytical study was performed, in which the 3-level EuroQol 5-dimensions (EQ-5D) questionnaire was used to measure the participant’s QoL and to estimate the quality of life determinants among insured individuals, including their socioeconomic factors and health status. The study was conducted among 600 people who had contacted a health insurance agency to buy new insurance or renew their previous insurance contract from May to June, 2016.

We sampled insured individuals in Tehran, Iran prior to the study. The study population consisted of all people who visited the two main insurance agencies, Social Health Insurance (SHI) and Iranian Health Insurance (IHI), who fulfilled the inclusion criteria of the study. All people who visited the SHI and IHI offices and who consented to participate in the study voluntarily were included. However, participants who were less than 18 years old or had cognitive problems were excluded from this study. A cluster sampling technique was used to collect data. For this purpose, Tehran was divided into northern, southern, western, eastern and central areas. One branch of the SSIO and IHIO was selected from each area. Finally, the participants were selected by convenience sampling. The number of participants in each branch was determined proportionally according to the insured population covered by each branch. The sampling ended when the target number of participants in each branch was reached.

### Instrument and measurement

An interviewer-administrated questionnaire was used to measure QoL and to identify the determinants of QoL among the insured. QoL was measured using the EQ-5D, which consists of items classified into five dimensions: mobility, self-care, usual activity, pain/discomfort, and anxiety/depression. Each item is answered using one of three responses: no problem, some problems, and extreme problems. The EQ-5D is a standard measurement scale for measuring QoL [2]. This study used the Iranian value set for EQ-5D health states in order to calculate participants’ QoL. This value set was derived using the visual analog scale method [16]. Socioeconomic variables are basic predictors of health and QoL [4,17]. Consequently, these variables and demographic variables were also included in this study.

### Statistical analysis

The analysis was performed using the descriptive characteristics of HRQoL status and insurance categories. Body mass index (BMI, kg/m^2^) was calculated by dividing the weight (in kilograms) of an individual by her/his height (in meters) squared [18]. We also categorized BMI into three groups using the World Health Organization standard cut-off points: normal range, BMI <25 kg/m^2^; overweight, 25 kg/m^2^ ≤BMI <30 kg/m^2^; and obese, BMI ≥30 kg/m^2^ [19]. We created a categorical variable including two types of health insurance combinations to evaluate whether insurance status modified the association between health insurance and HRQoL. The QoL scores were categorized into three groups: low (QoL<0.5), moderate (0.5≤QoL<0.8), and high (QoL≥0.8). Furthermore, in order to test the hypothesis that QoL scores were independent of other variables (sex, age, etc.), the chi-square test was performed. Variables such as age and years of schooling were categorized. However, for variables such as marital status, employment status, and type of health insurance, some groups were merged to meet the assumptions of the chi-square test [20]. Consequently, regression analysis was performed to estimate the effects of the variables in a single model. Heteroscedasticity was found in the model. Therefore, weighted least squares regressions were constructed to model HRQoL measures in relation to HRQoL and insurance status. Therefore, variables that had a significant dependency with QoL using the chi-square test were included in the regression model. The Ramsey Regression Equation Specification Error Test showed that there were no omitted variables. The final model was as follows:

$$QoL\;=\;\beta_{0\;}+\;\beta_{1}\;sex\;+\;\beta_{2}\;age\;+\;\beta_{3}\;mari\;+\;\beta_{4}\;ysch\;+\;\beta_{5}\;inc\;+\;\beta_{6\;}emp\;+\;\beta_{7\;}chd\;+\;\beta_{8}\;BMI$$

where *sex* refers to sex, *age* to age, *mari* to marital status, *ysch* to years of schooling, *inc* to income, *emp* to employment status, *chd* to chronic disease(s), and *BMI* to body mass index. We used Stata version 11.0 (StataCorp, College Station, TX, USA) for all statistical analyses in the study.

## RESULTS

A total of 600 people with insurance participated in the study. The response rate was 93.5%. 327 participants (54.5%) were male and 273 (45.5%) were female. The mean age of participants was 41.48 years (standard deviation [SD], 14.60 years). The mean duration of education was 12.36 years (SD, 4.68 years). The mean BMI was 25.75 kg/m^2^ (SD, 4.20 kg/m^2^). The majority of participants (n=379, 63.2%) were married, while 164 (27.3%) were single and the remaining 57 (9.5%) were divorced or bereaved. Participants with moderate QoL were more likely to be married than single. However, single participants showed a similar QoL (both moderate and high). Of the participants, 300 (50.0%) were heads of households.

A total of 446 participants (74.3%) were insured through SSI, while 154 (25.7%) were ensured through other health insurance agencies, of whom 119 (19.8%) had insurance through IHI. Furthermore, we found that the majority of the insured (n=392, 65.3%) did not have complementary health insurance. Of the participants, 316 (52.6%) were employed.

Most participants (n=306, 51.0%) earned <20 million Iranian rials (IRR) monthly, and 189 (31.5%) earned between 20 and 40 million IRR. Only 105 (17.5%) earned 40 million IRR or more monthly. Meanwhile, 95 participants (15.8%) were current smokers and 338 (56.3%) were affected by chronic diseases in the study period. The mean HRQoL score was 0.74 (SD, 0.16).

The overall QoL was most commonly moderate (52.7% of participants). Statistically significant differences in QoL were found according to sex, age, marital status, years of schooling, employment status, monthly income, chronic disease(s), and BMI (p<0.001). However, no significant differences in QoL were found according to head of household status (p=0.52), insurance type (p=0.85), complementary insurance (p=0.48), and smoking status (p=0.45). It was also found that among individuals with moderate QoL score (0.5 to 0.8), 171 (54.1%) were male and 145 (45.9%) were female. In addition, the majority (n=232, 73.4%) of participants with moderate QoL score were insured by SSI and the remaining 84 (26.6%) were insured by other insurance agencies.

Almost all of the variables that were related to QoL were associated with moderate QoL scores. However, 47 (44.8%) of the insured who earned at least IRR 40 million monthly salary had high QoL scores (≥0.80), and 178 (67.9%) of the insured who are not affected by chronic diseases had a high QoL score (≥0.80) (Table 1).

The EQ-5D indicated that respondents 50 to 59 years of age reported poorer health status. Respondents in this age group reported the highest proportion of problems with anxiety/depression (44.2%). The proportion of respondents self-reporting their EQ-5D profile to be level 3 (extreme problems) was 0.0% in all age groups for the mobility, self-care and, usual activity dimensions. Respondents 18 to 29 years of age had the lowest proportion of individuals reporting problems with self-care (0.7%), and the age group of 30 to 39 years had the lowest percentage of problems in the mobility dimension (18.8%). The age group of 40 to 49 years had the lowest percentage of usual activity problems (9.0%) (Table 2).

**Figure 1.**
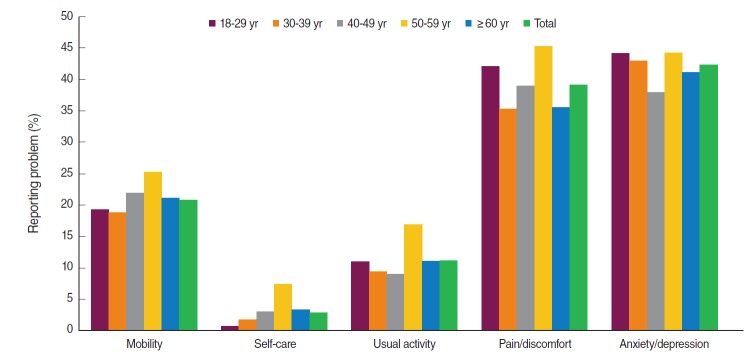
Percentage distribution of reporting problems among health Insured in Tehran city, Iran, 2016.

Compared with the other age groups, the age group of 50 to 59 years reported significantly greater problems in the mobility, self-care, usual activity, and pain/discomfort dimensions of the EQ-5D. No significant differences were found in the proportion of participants who reported problems in the usual activity and anxiety/depression dimensions between 18 and 49 years of age. Compared with the overall rate of problems reported in each age groups, a significantly higher proportion of participants reported problems in the anxiety/depression dimension. Therefore, the highest percentage of participants who reported problems in the pain/discomfort (45.2%) and anxiety/depression (44.2%) dimensions were in the 50 to 59 years-old age group. Overall, participants 50 to 59 years of age and ≥60 years of age had more problems in all EQ-5D dimensions (Figure 1).

The results of regression analysis predicting the eight outcome variables after controlling for the characteristics of the insured found that the average QoL score in females was 0.047 lower than in males (p<0.001). The variables showing the strongest associations were sex, years of schooling, and chronic diseases. Marital status, income (in the category of IRR 20 to 40 million monthly) and employment status were not significantly related to QoL. However, the variables showing slight associations with QoL were age, marital status, income, BMI, and employment status. The healthy insured had a QoL score 0.119 greater than people with a chronic disease (p<0.001). Moreover, each unit increase of BMI yielded a 0.004 unit decrease in QoL (p<0.05). The R2 of the model was 0.32 and the model was statistically significant (p<0.001) (Table 3).

## DISCUSSION

This study measured HRQoL and determined the effects of factors related to HRQoL, using the EQ-5D to measure outcomes among individuals with basic insurance and their QoL. We found that the greatest proportion of problems such as anxiety/depression was reported by individuals 50 years of age and older.

This study is supported by a previous study [16] showing that pain/discomfort was the most common health problem (34.4%) and that 33.4% of the population suffered from anxiety/depression in Tehran. Similar studies have shown that most problems in the general population related to the pain/discomfort and anxiety/depression dimensions of HRQoL. This study revealed that individuals with insurance aged 18 to 44 years had the lowest proportion of problems with mobility, self-care, and usual activity. Similarly, self-care was the least prevalent health problem in these subjects [4,21,22].

This study suggested that HRQoL significantly varied with sex. The mean EQ-5D values in Sweden (0.85) [4], in the US (0.87) [23] and in Italy (0.96) [22] were higher than our study (0.74). The SD of the scores in all these studies was lower than ours, as well. Other studies [22,24] have shown that QoL for females is lower than for males. Moreover, with increasing age, QoL decreases. Another study [25] that investigated HRQoL in the elderly population in Tehran showed that elderly individuals suffered from poor QoL. Furthermore, females and lower education levels were associated with lower HRQoL. In many studies, people with higher income and education reported higher HRQoL scores [4,24,26,27]. Nonetheless, some studies did not report a significant difference in QoL based on sexes [21]. Our results also showed that no significant difference was found in QoL according to insurance type, complementary insurance, or smoking status. Similar to our study, the causal relationship between income and health status was not clear, but this information may be very valuable for policymakers nonetheless [24].

Some evidence suggests that QoL is negatively affected by smoking [22]. Additionally, patients with chronic diseases who smoke have a lower health status than never smokers [28].

This study provides evidence that QoL was strongly associated with monthly income, chronic diseases, and BMI. High BMI had significant negative effects on HRQoL. However, a previous study [4] showed that QoL scores were not correlated with marital status or employment patterns. Therefore, it seems that more studies are needed to clarify the correlation between these variables and QoL. Similarly, many studies have shown the negative impact of excess body weight on QoL [29,30]. Additionally, some other studies have shown that obese people who do not have chronic diseases have a lower QoL than people with a normal weight [31]. It has previously been reported that adults with a higher than normal BMI have poorer physical and mental QoL than people with normal weight and that people with a higher class of obesity have poorer QoL [29,30]. A national survey in Iran [15] showed that people with type 2 diabetes, as a prevalent chronic disease, had poor HRQoL, especially in the pain/discomfort and anxiety/depression dimensions of HRQoL.

Our findings indicate that the overall QoL among the insured was moderate. In addition, this study revealed that sex, years of schooling, and chronic diseases were strongly associated with QoL. A similar study [24] showed that females, lower education, unemployment, and hospitalization due to diabetes and side effects of the disease were correlated with lower QoL and reporting some or extreme problems in various dimensions. Moreover, the difference in QoL scores between people with and without any disease was approximately 0.064, which is less than what we found (0.119). Another study [32] also indicated that patients with coronary heart disease in both sexes had lower QoL scores than the general population and a higher percentage of reported problems in the anxiety/depression dimension of EQ-5D. In addition, some evidence shows that people with chronic conditions have poorer physical and mental QoL [33], and that those with more severe disease have poorer QoL [34]. However, therapeutic interventions may have a significant impact on improving QoL in these patients [33,34].

We used convenience sampling for data gathering and it was not possible to select participants randomly. Additionally, participants were asked to provide their weight and height to calculate their BMI. Therefore, it is possible that some errors occurred in this variable.

The findings of our study contribute to improved perceptions of individuals’ own health status and QoL in Iran. The attention of policymakers, insured individuals, and communities in general should be focused on the effects of the factors affecting insurance status and HRQoL. Moreover, chronic diseases are the highest priority for interventions to improve the health status of individuals with insurance. This evidence can help policymakers and health insurance organizations to improve their planning to promote HRQoL among insured individuals and society as a whole.

## Figures and Tables

**Table 1.** Variations in quality of life (QoL) among individuals with health insurance in Tehran, Iran, 2016

	QoL				p-value
	Low	Moderate	High	Total	
Overall	51 (8.5)	316 (52.7)	233 (38.8)	600	
Sex					
Male	13 (4.0)	171 (52.3)	143 (43.7)	327	<0.001
Female	38 (13.9)	145 (53.1)	90 (33.0)	273	
Age (yr)					
18-29	2 (1.4)	64 (44.1)	79 (54.5)	145	<0.001
30-39	8 (4.7)	83 (48.8)	79 (46.5)	170	
40-49	14 (14.0)	60 (60.0)	26 (26.0)	100	
50-59	15 (15.8)	53 (55.8)	27 (28.4)	95	
≥60	12 (13.3)	56 (62.2)	22 (24.4)	90	
Marital status					
Single	5 (3.0)	80 (48.8)	79 (48.2)	164	<0.001
Married	32 (8.4)	199 (52.5)	148 (39.0)	379	
Other (divorced/bereaved)	14 (24.6)	37 (64.9)	6 (10.5)	57	
Head of household					
Yes	22 (7.3)	163 (54.3)	115 (38.3)	300	0.52
No	29 (9.7)	153 (51.0)	118 (39.3)	300	
Insurance type					
SSI	38 (8.5)	232 (52.0)	176 (39.5)	446	0.85
IHI/other	13 (8.4)	84 (54.5)	57 (37.0)	154	
Complementary insurance					
Yes	14 (6.7)	114 (54.8)	80 (38.5)	208	0.48
No	37 (9.4)	202 (51.5)	153 (39.0)	392	
Chronic disease(s)					
Yes	46 (13.6)	237 (70.1)	55 (16.3)	338	<0.001
No	5(1.9)	79 (30.1)	178 (67.9)	262	
Smoking					
Yes	5 (5.7)	53 (55.8)	37 (38.9)	95	0.45
No	46 (9.1)	263 (52.1)	196 (38.8)	505	
Years of schooling					
<9	22 (19.0)	65 (56.0)	29 (25.0)	116	<0.001
9-14	23 (10.3)	115 (51.6)	85 (38.1)	223	
≥14	6 (2.3)	136 (52.1)	119 (45.6)	261	
Employment status					
Employed	12 (3.8)	161 (50.9)	143 (45.2)	316	0.001
Other (unemployed, housekeeper, retired, etc.)	39 (13.7)	155 (54.6)	90 (31.7)	284	
Income (million IRR)					
<20	42 (13.7)	154 (50.3)	110 (35.9)	306	<0.001
20-40	9 (2.9)	104 (34)	76 (24.8)	189	
≥40	0 (0)	58 (18.9)	47 (15.4)	105	
BMI (kg/m^2^)					
< 25	16 (5.9)	136 (50.4)	118 (43.7)	270	<0.001
25-30	16 (6.6)	134 (55.4)	92 (38.0)	242	
≥30	19 (21.6)	46 (52.3)	23 (26.1)	88	

Values are presented as number (%). SSI, Social Security Insurance; IHI, Iranian Health Insurance; IRR, Iranian rial; BMI, body mass index.

**Table 2.** Proportions of level 1, 2, and 3 responses by dimension of the EuroQol 5-dimensions (EQ-5D) and by age group among individuals with health insurance in Tehran, Iran, 2016

EQ-5D		Age (yr)					
	Level	18-29	30-39	40-49	50-59	≥60	Total
Mobility	1	80.7	81.1	78.0	74.7	78.9	79.2
	2	19.3	18.8	22.0	25.3	21.1	20.8
	3	0.0	0.0	0.0	0.0	0.0	0.0
Self-care	1	99.3	98.2	97.0	92.6	96.7	97.2
	2	0.7	1.8	3.0	7.4	3.3	2.8
	3	0.0	0.0	0.0	0.0	0.0	0.0
Usual activity	1	89.0	90.6	91.0	83.2	88.9	88.8
	2	11.0	9.4	9.0	16.8	11.1	11.2
	3	0.0	0.0	0.0	0.0	0.0	0.0
Pain/discomfort	1	57.9	64.7	61.0	54.7	64.4	60.8
	2	40.7	35.3	39.0	42.1	34.4	38.2
	3	1.4	0.0	0.0	3.2	1.1	1.0
Anxiety/depression	1	55.9	57.1	62.0	55.8	58.9	57.7
	2	39.3	38.8	37.0	38.9	40.0	38.8
	3	4.8	4.1	1.0	5.3	1.1	3.5

**Table 3.** Regression analysis results of quality of life among individuals with health insurance in Tehran, Iran, 2016

	β-coefficient	Robust SE	p-value	95% CI	
				Upper limit	Low limit
Sex (ref: male)					
Female	-0.047	0.013	< 0.001	-0.072	-0.021
Age (ref: 18-29, yr)					
30-39	-0.013	0.013	0.33	-0.039	0.013
40-49	-0.050	0.019	0.01	-0.089	-0.012
50-59	-0.042	0.021	0.05	-0.084	0.000
≥60	-0.046	0.023	0.04	-0.091	-0.001
Marital status (ref: single)					
Married	0.012	0.013	0.34	-0.013	0.037
Divorced/ bereaved	-0.024	0.029	0.40	-0.081	0.032
Years of schooling (ref: 0-8)					
9-13	0.030	0.020	0.14	-0.010	0.069
14+	0.050	0.018	0.007	0.014	0.086
Income (ref: <20 million IRR)					
20-40	0.023	0.013	0.07	-0.002	0.048
≥40	0.035	0.015	0.02	0.005	0.066
Employment status (ref: employed)					
Other (unemployed, housekeeper, retired, etc.)	-0.010	0.013	0.44	-0.035	0.015
Chronic disease(s) (ref: yes)					
No	0.119	0.011	< 0.001	0.097	0.141
BMI	-0.004	0.001	0.03	-0.007	-0.000
Constant	0.792	0.047	< 0.001	0.699	0.884
R-squared = 0.32 F (12, 586) = 19.17 Prob>F = 0.000					

SE, standard error; CI, confidence interval; IRR, Iranian rial; BMI, body mass index.
